# Acute cholecystitis in old adults: the impact of advanced age on the clinical characteristics of the disease and on the surgical outcomes of laparoscopic cholecystectomy

**DOI:** 10.1186/s12876-023-02954-6

**Published:** 2023-09-25

**Authors:** Cho Eun Lee, Seung Jae Lee, Ju Ik Moon, In Seok Choi, Dae Sung Yoon, Won Jun Choi, Sang Eok Lee, Nak Song Sung, Seong Uk Kwon, In Eui Bae, Seung Jae Roh, Sung Gon Kim

**Affiliations:** Department of Surgery, Konyang University Hospital, Konyang University College of Medicine, 158, Gwanjeodong-ro, Seo-gu, 35365 Daejeon, South Korea

**Keywords:** Acute cholecystitis, Laparoscopy, Cholecystectomy, Aged, 80 and over

## Abstract

**Background:**

Impact of advanced age on disease characteristics of acute cholecystitis (AC), and surgical outcomes after laparoscopic cholecystectomy (LC) has not been established.

**Methods:**

This single-center retrospective study included patients who underwent LC for AC between April 2010 and December 2020. We analyzed the disease characteristics and surgical outcomes according to age: Group 1 (age < 60 years), Group 2 (60 ≤ age < 80 years), and Group 3 (age ≥ 80 years). Risk factors for complications were assessed using logistic regression analysis.

**Results:**

Of the 1,876 patients (809 [43.1%] women), 723 were in Group 1, 867 in Group 2, and 286 in Group 3. With increasing age, the severity of AC and combined common bile duct stones increased. Group 3 demonstrated significantly worse surgical outcomes when compared to Group 1 and 2 for overall (4.0 vs. 9.1 vs. 18.9%, *p* < 0.001) and serious complications (1.2 vs. 4.2 vs. 8.0%, *p* < 0.001), length of hospital stay (2.78 vs. 3.72 vs. 5.87 days, *p* < 0.001), and open conversion (0.1 vs. 1.0 vs. 2.1%, *p* = 0.007). Incidental gallbladder cancer was also the most common in Group 3 (0.3 vs. 1.5 vs. 3.1%, *p* = 0.001). In the multivariate analysis, body mass index < 18.5, moderate/severe AC, and albumin < 2.5 g/dL were significant risk factors for serious complications in Group 3.

**Conclusion:**

Advanced age was associated with severe AC, worse surgical outcomes, and a higher rate of incidental gallbladder cancer following LC. Therefore, in patients over 80 years of age with AC, especially those with poor nutritional status and high severity grading, urgent surgery should be avoided, and surgery should be performed after sufficient supportive care to restore nutritional status before LC.

## Background

Acute cholecystitis (AC) is one of the most common surgical indications of gastrointestinal diseases. Cholecystectomy is a treatment of choice for AC, and currently, laparoscopic cholecystectomy (LC) is the golden standard on AC [1, 2].

The most common cause of AC is gallstones, the prevalence and complications of which have been shown to increase with age [3, 4]. As life expectancy has increased, the incidence of AC has also increased. Although LC is considered relatively safe, a 6–9% risk of serious complication and 0.1–0.3% of mortality still exist [5, 6]. Advanced age may be associated with a high risk of postoperative complications owing to greater comorbidities and poorer general conditions [7]. Percutaneous transhepatic gallbladder drainage (PTGBD) is considered an alternative treatment option for high-risk older patients. However, we previously reported that elective LC is better than conservative treatment in older patients with AC [8]. Therefore, the treatment strategy for AC in older patients remains controversial.

Recently, some studies have reported poor surgical outcomes for LC in older patients [9, 10]. Nevertheless, data on clinical characteristics of the disease and on the surgical outcomes of LC in elderly patients with AC are still lacking. In addition, treatment outcomes of AC can differ from clinical features, such as the severity of AC and combined common bile duct (CBD) stones [11]. Therefore, the aim of this study is to establish the impact of advanced age on disease characteristics of AC and surgical outcomes after LC.

## Methods

### Patients

From April 2010 to December 2020, all consecutive patients diagnosed with AC who underwent elective LC at Konyang University Hospital were evaluated. Patients with suspected gallbladder cancer, based on imaging studies, or those who underwent emergency LC and other combined surgeries were excluded. A total of 1876 patients were included in the study. The diagnosis and classification of AC severity were based on the 2018 Tokyo Guidelines [12]. We divided the study population into three groups according to age and retrospectively reviewed the patients’ demographics, disease characteristics, and surgical outcomes: Group 1 (age < 60 years), Group 2 (60 ≤ age < 80 years), and Group 3 (age ≥ 80 years).

This study was approved by the Institutional Review Board of Konyang University Hospital (IRB No. 2022-01-016), and the requirement for obtaining informed consent was waived owing to the retrospective study design by the Institutional Review Board of Konyang University Hospital. All methods were performed in accordance with the relevant guidelines and regulations.

### Definition of demographics and surgical outcomes

The general condition and physical fitness of each patient were evaluated using the Charlson Age Comorbidity Index (CACI) [13] and American Society of Anesthesiologists physical status (ASA PS) classification [14]. The nutritional status of the patients was assessed using body mass index (BMI) and preoperative serum albumin level. The presence of gallstones and CBD stones was confirmed by imaging studies using abdominal ultrasonography, computed tomography, or magnetic resonance cholangiopancreatography. PTGBD is indicated in patients with AC who are not fit for immediate surgery due to the high risk of surgery at the time of presentation. The detailed indication for PTGBD has been described in our previous study [15]. When PTGBD was performed, following LC was performed within the same hospitalization period. Operation time was calculated as the time from skin incision to skin closure. Blood loss estimates were obtained from surgical records. Subtotal cholecystectomy was defined as making an incision in the gallbladder, aspirating the contents, and removing as much of the gallbladder wall as possible, with the aim of treating the stump instead of removing the entire gallbladder [16].

Postoperative complications were graded according to the Clavien-Dindo classification [17]. Adjacent organ injury was defined as unintended damage requiring repair of organs other than the gallbladder, such as the bile duct, hepatic artery, duodenum, small intestine, and colon. Serious complications were defined as a level greater than grade III of the Clavien-Dindo classification. The postoperative hospital stay was defined as the number of days of hospital stay after LC. The total hospital stay was defined as the number of days between admission and discharge. Pulmonary complications were defined as any complication affecting the respiratory system after general anesthesia and LC, including pneumonia, atelectasis, pleural effusion, and respiratory failure [18]. The diagnosis of bile leakage was based on the definition provided by the International Study Group of Liver Surgery [19].

### Statistical analysis

Continuous variables were summarized as mean and standard deviation and compared using analysis of variance. Categorical variables are presented as counts and percentages and were compared using the Chi-square test. Univariate and multivariate analyses were performed to identify the predictors of serious complications after LC in patients of advanced age. Multivariate analyses of the significant factors identified in univariate analyses were performed using a logistic regression model. All tests were two-sided, and p-values less than 0.05 were considered statistically significant. Statistical analyses were performed using SPSS version 27 (SPSS Inc., Chicago, IL, USA).

## Results

### Patients’ characteristics

The demographic and disease characteristics of the study population are shown in Table 1. A total of 1876 patients were divided into three groups according to age: Group 1 (age < 60 years, n = 723), Group 2 (60 ≤ age < 80 years, n = 867), and Group 3 (age ≥ 80 years, n = 286). Female patients were most common in group 3 (45.0% vs. 37.4% vs. 55.9%, *p* < 0.001). With increasing age, BMI decreased (25.5 kg/m^2^ vs. 24.7 kg/m^2^ vs. 23.0 kg/m^2^, *p* < 0.001), and ASA PS (≥ III; 6.1% vs. 33.9% vs. 60.8%, *p* < 0.001), CACI (≥ 6; 0.0% vs. 3.6% vs. 27.6%, *p* < 0.001), severity of AC (grade III; 0.4% vs. 4.5% vs. 8.7%, *p* < 0.001), acalculous cholecystitis (22.7% vs. 29.5% vs. 35.0%, *p* < 0.001), and combine CBD stones (15.6% vs. 19.5% vs. 24.8%, *p* < 0.001) increased. There was no significant difference in previous abdominal surgery history among the three groups. In laboratory findings, there were significant differences between the three groups in WBC, hemoglobin, platelet, PT INR, creatinine, albumin, total bilirubin, and CRP levels; however, there were no significant differences in AST and ALT levels.

**Table 1:** Comparison of patients’ characteristics according to the age

Variable	Total (n = 1876)	Age < 60 (n = 723)	60 ≤ Age < 80 (n = 867)	Age ≥ 80 (n = 286)	P-value
Age, mean years (SD)	62.7 (16.2)	45.6 (9.9)	69.9 (5.7)	84.0 (3.6)	< 0.001
Female, n (%)	809 (43.1)	325 (45.0)	324 (37.4)	160 (55.9)	< 0.001
BMI, mean kg/m^2^ (SD)	24.8 (3.6)	25.5 (3.8)	24.7 (3.2)	23.0 (3.4)	< 0.001
Charlson age comorbidity index, n (%)					< 0.001
0–5 ≥ 6	1766 (94.1) 110 (5.9)	723 (100.0) 0 (0.0)	836 (96.4) 31 (3.6)	207 (72.4) 79 (27.6)	
ASA PS classification, n (%)					< 0.001
1–2	1364 (72.7)	679 (93.9)	573 (66.1)	112 (39.2)	
3–5	512 (27.3)	44 (6.1)	294 (33.9)	174 (60.8)	
Previous abdominal surgery (+), n (%)	336 (17.9)	137 (18.9)	151 (17.4)	48 (16.8)	0.631
Severity according to the TG 18, n (%)					< 0.001
Grade I	1374 (73.2)	611 (84.5)	592 (68.3)	171 (59.8)	
Grade II	435 (23.2)	109 (15.1)	236 (27.2)	90 (31.5)	
Grade III	67 (3.6)	3 (0.4)	39 (4.5)	25 (8.7)	
Acalculous cholecystitis on preoperative imaging, n (%)	520 (27.7)	164 (22.7)	256 (29.5)	100 (35.0)	< 0.001
Combined CBD stone on preoperative imaging, n (%)	353 (18.8)	113 (15.6)	169 (19.5)	71 (24.8)	0.003
Preoperative laboratory findings					
WBC, mean 10^3^/mm3 (SD)	11.7 (5.2)	10.9 (4.5)	12.2 (5.6)	12.6 (5.4)	< 0.001
Hemoglobin, mean g/dL (SD)	13.4 (1.7)	13.9 (1.7)	13.3 (1.6)	12.5 (1.8)	< 0.001
Platelet, mean 103/mm3 (SD)	223.8 (72.3)	242.7 (66.0)	211.9 (72.9)	211.8 (76.1)	< 0.001
PT, mean INR (SD)	1.11 (0.17)	1.07 (0.12)	1.12 (0.15)	1.16 (0.28)	< 0.001
Creatinine, mean mg/dL (SD)	0.95 (0.61)	0.82 (0.52)	1.01 (0.67)	1.07 (0.58)	< 0.001
Albumin, mean g/dL (SD)	3.70 (0.58)	4.01 (0.48)	3.59 (0.54)	3.21 (0.47)	< 0.001
AST, mean IU/L (SD)	129.8 (273.9)	119.1 (247.1)	132.2 (300.4)	149.6 (252.7)	0.263
ALT, mean IU/L (SD)	104.7 (191.1)	115.1 (201.2)	99.8 (197.0)	93.1 (138.7)	0.152
Total bilirubin, mean mg/dL (SD)	1.77 (1.83)	1.60 (1.74)	1.87 (1.94)	1.88 (1.69)	0.007
CRP, mean mg/dL (SD)	8.68 (10.34)	5.98 (8.98)	9.88 (10.70)	11.42 (10.84)	< 0.001
Preoperative PTGBD, n (%)	1020 (54.4)	271 (37.5)	529 (61.0)	220 (76.9)	< 0.001

SD: standard deviation; BMI, body mass index; ASA PS: American Society of Anesthesiologists physical status; TG 18: Tokyo guideline 2018; CBD: common bile duct; WBC: white blood cell; PT INR: prothrombin time international normalized ratio; AST: aspartate aminotransferase; ALT: alanine aminotransferase; CRP: C-reactive protein; PTGBD: percutaneous transhepatic gallbladder drainage

A total of 1876 patients, preoperative PTGBD was performed in 1020 (54.4%) with a median interval from PTGBD to LC of 5 days (minimum 1 and maximum 64. of interval days). Preoperative PTGBD was most frequently performed in Group 3 (37.5% vs. 61.0% vs. 76.9%, *p* < 0.001).

### Surgical outcomes

A comparison of the surgical variables according to age is presented in Table 2. There were no significant differences in operation time, adjacent organ injury detected during surgery, or subtotal cholecystectomy. With increasing age, estimated blood loss (19.4 mL vs. 26.6 mL vs. 28.9 mL, *p* = 0.028), open conversion (0.1% vs. 1.0% vs. 2.1%, *p* = 0.007), overall complication (4.0% vs. 9.1% vs. 18.9%, *p* < 0.001), serious complication (1.2% vs. 4.2% vs. 8.0%, *p* < 0.001), 90-day mortality (0.0% vs. 0.1% vs. 2.1%, *p* < 0.001), postoperative hospital stay (2.02 days vs. 3.72 days vs. 5.87 days, *p* < 0.001), total hospital stay (3.76 days vs. 6.64 days vs. 10.69 days, *p* < 0.001), surgical site infection (1.5% vs. 4.2% vs. 6.3%, *p* < 0.001), pulmonary complication (0.6% vs. 2.5% vs. 6.6%, *p* < 0.001), and re-admission (0.3% vs. 0.8% vs. 1.7%, *p* = 0.048) increased.

**Table 2:** Comparison of surgical outcomes according to the age

Variable	Total (n = 1876)	Age < 60 (n = 723)	60 ≤ Age < 80 (n = 867)	Age ≥ 80 (n = 286)	P-value
Operation time, mean minutes (SD)	60.0 (27.0)	58.9 (26.7)	60.2 (25.6)	62.1 (31.4)	0.238
Estimated blood loss, mean mL (SD)	24.2 (62.7)	19.4 (51.6)	26.6 (69.0)	28.9 (67.6)	0.028
Adjacent organ injury detected during surgery, n (%)	22 (1.2)	5 (0.7)	11 (1.3)	6 (2.1)	0.163
Bile duct	8 (0.4)	3 (0.4)	3 (0.3)	2 (0.7)	
Duodenum	2 (0.1)	1 (0.1)	1 (0.1)	0 (0.0)	
Colon	2 (0.1)	0 (0.0)	1 (0.1)	1 (0.3)	
Hepatic artery	6 (2.2)	1 (0.1)	3 (0.3)	2 (0.7)	
Small intestine	4 (0.2)	0 (0.0)	3 (0.3)	1 (0.3)	
Open conversion, n (%)	16 (0.9)	1 (0.1)	9 (1.0)	6 (2.1)	0.007
Subtotal cholecystectomy, n (%)	10 (0.5)	4 (0.6)	4 (0.5)	2 (0.7)	0.888
Overall complication, n (%)	162 (8.6)	29 (4.0)	79 (9.1)	54 (18.9)	< 0.001
Serious complication (≥ C-D classification III), n (%)	68 (3.6)	9 (1.2)	36 (4.2)	23 (8.0)	< 0.001
90-day mortality, n (%)	7 (0.4)	0 (0.0)	1 (0.1)	6 (2.1)	< 0.001
Postoperative hospital stay, mean days (SD)	4.26 (25.88)	2.78 (2.02)	3.72 (5.17)	5.87 (9.22)	< 0.001
Total hospital stay, mean days (SD)	9.84 (26.36)	6.95 (3.76)	9.87 (6.64)	13.19 (10.69)	< 0.001
Surgical site infection, n (%)	65 (3.5)	11 (1.5)	36 (4.2)	18 (6.3)	< 0.001
Bile leakage, n (%)	12 (0.6)	2 (0.3)	7 (0.8)	3 (1.0)	0.268
Pulmonary complication, n (%)	45 (2.4)	4 (0.6)	22 (2.5)	19 (6.6)	< 0.001
Re-operation, n (%)	10 (0.5)	2 (0.3)	5 (0.6)	3 (1.0)	0.307
Re-admission, n (%)	14 (0.7)	2 (0.3)	7 (0.8)	5 (1.7)	0.048
Incisional hernia, n (%)	8 (0.4)	0 (0.0)	6 (0.7)	2 (0.7)	0.081
Incidental gallbladder cancer, n (%)	24 (1.3)	2 (0.3)	13 (1.5)	9 (3.1)	0.001

SD: standard deviation; C-D classification, Clavien-Dindo classification

In the univariate analysis, age ≥ 80 years was a statistically significant risk factor for incidental gallbladder cancer after LC (odds ratio [OR] 11.713, *p* = 0.002) (Table 3).

**Table 3:** Univariate analysis of age-related risk for incidental gallbladder cancer

Factor		Univariate analysis	
		OR (95% CI)	P-value
Age			
< 60		1 (ref)	-
≥ 60 & < 80		5.488 (1.234–24.398)	0.025
≥ 80		11.713 (2.515–54.550)	0.002

OR: odds ratio; CI: confidence interval

### Details of the serious postoperative complications

Serious postoperative complications were classified according to the Clavien-Dindo classification, and 65 were reported (Table 4). The percentage of serious complications (1.2% vs. 4.2% vs. 8.0%) increased with advancing age. In grade IIIa complications, fluid collection with percutaneous drain (PCD) insertion was the most common, followed by bile leakage with endoscopic nasobiliary drainage or endoscopic retrograde biliary drainage insertion, pleural effusion with PCD insertion, CBD stone with endoscopic stone extraction, and wound dehiscence with local repair. Re-operation included mechanical ileus, bile leakage, wound dehiscence, pressure sore, and empyema of the lung, and was reported as grade IIIb. With increasing age, grade IV and V complications increased (Grade IV, 0.1% vs. 0.7% vs. 2.4%); Grade V, 0.0% vs. 0.1% vs. 2.0%). Among grade IV complications, pneumonia with mechanical ventilation was the most common, followed by mechanical ileus with re-operation and cerebrovascular accident with ICU care. Among grade V complications, five cases of pneumonia and two cases of organ space surgical site infection have been reported.

**Table 4:** Postoperative serious complications based on the Clavien-Dindo classification according to the age of patients

Clavien-Dindo classification	Age < 60 (n = 723)	60 ≤ Age < 80 (n = 867)	Age ≥ 80 (n = 286)
Grade IIIa, n (%)	5 (0.7)	27 (3.1)	9 (3.1)
Fluid collection with PCD insertion	2	14	4
Bile leakage with ENBD/ERBD insertion	0	7	3
CBD stone with endoscopic stone extraction	2	0	0
Pleural effusion with PCD insertion	1	5	2
Wound dehiscence with local repair	0	1	0
Grade IIIb, n (%)	3 (0.4)	2 (0.2)	1 (0.3)
Mechanical ileus with re-operation	1	0	0
Bile leakage with re-operation	1	0	0
Wound dehiscence with re-operation	1	0	1
Pressure sore with re-operation	0	1	0
Empyema of the lung with re-operation	0	1	0
Grade IV, n (%)	1 (0.1)	6 (0.7)	7 (2.4)
Bile leakage with ENBD insertion + ICU care	1	0	0
Mechanical ileus with re-operation + ICU care	0	1	1
Pneumonia with mechanical ventilator	0	2	4
Fluid collection with PCD + ICU care	0	1	0
Fluid collection with re-operation + ICU care	0	1	0
Cerebrovascular accident + ICU care	0	1	1
Hernia strangulation with re-operation + ICU care	0	0	1
Grade V, n (%)	0 (0.0)	1 (0.1)	6 (2.0)
Pneumonia	0	1	4
Organ space surgical site infection	0	0	2
Total, n (%)	9 (1.2)	36 (4.2)	23 (8.0)

PCD: percutaneous drainage; ENBD: endoscopic nasobiliary drainage; ERBD: endoscopic retrograde biliary drainage; CBD: common bile duct; ICU: intensive care unit

### Risk factor for serious complications in patients over 80 years of age

The results of univariate and multivariate analyses for serious complications (Clavien-Dindo classification grade III to V) in patients aged > 80 years are presented in Table 5. Univariate analysis revealed that low BMI (< 18.5 kg/m^2^), severity of AC (moderate and severe AC), and low serum albumin (< 2.5 g/dL) were statistically significant. In multivariate analysis, low BMI (< 18.5 kg/m^2^, OR 4.254, *p* = 0.019), severity of AC (moderate, OR 3.545, *p* = 0.015; severe, OR 3.924, *p* = 0.048), and low serum albumin (> 2.5 g/dL, OR 4.414, *p* = 0.032) were identified as significant risk factors for serious complications after LC in older patients.

**Table 5:** Univariate and multivariate analyses of risk factors for serious complications in patients over 80 years of age

Factor	Multivariate analysis		Multivariate analysis	
	OR (95% CI)	P-value	OR (95% CI)	P-value
Gender				
Female	1 (ref)			
Male	1.726 (0.730–4.077)	0.214		
Charlson age comorbidity index				
< 7	1 (ref)			
≥ 7	1.520 (0.486–4.751)	0.472		
Body mass index, kg/m^2^				
> 20	1 (ref)		1 (ref)	
18.5–20.0	1.095 (0.302–3.965)	0.890	-	
< 18.5	4.078 (1.322–12.580)	0.014	4.254 (1.273–14.221)	0.019
ASA PS classification				
< 3	1 (ref)			
≥ 3	1.226 (0.502–2.995)	0.654		
Previous abdominal surgery				
No	1 (ref)			
Yes	1.421 (0.501–4.034)	0.509		
Severity of acute cholecystitis				
Mild	1 (ref)		1 (ref)	
Moderate	3.604 (1.366–9.512)	0.010	3.545 (1.282–9.807)	0.015
Severe	4.463 (1.204–16.535)	0.025	3.924 (1.012–15.211)	0.048
WBC, 10^3^/mm3				
< 18.0	1 (ref)			
≥ 18.0	0.862 (0.244–3.038)	0.817		
Albumin, g/dL				
≥ 2.5	1 (ref)		1 (ref)	
< 2.5	6.711 (1.852–24.318)	0.004	4.414 (1.136–17.153)	0.032
Total bilirubin, mg/dL				
< 2.0	1 (ref)			
≥ 2.0	1.019 (0.403–2.574)	0.968		
Creatinine, mg/dL				
< 2.0	1 (ref)			
≥ 2.0	3.436 (0.886–13.327)	0.074		
CRP, mg/dL				
< 20.0	1 (ref)			
≥ 20.0	1.795 (0.685–4.702)	0.234		
EST				
No	1 (ref)			
Yes	0.268 (0.061–1.172)	0.080		
PTGBD				
No	1 (ref)			
Yes	0.661 (0.260–1.683)	0.385		

OR: odds ratio; CI: confidence interval; ASA PS: American Society of Anesthesiologists physical status; WBC: white blood cell; CRP: C-reactive protein; EST: endoscopic sphincterotomy; PTGBD: percutaneous transhepatic gallbladder drainage

## Discussion

AC is an inflammatory disease of the gallbladder caused by impacted gallstone obstruction of the outlet of the gallbladder, either in the infundibulum or in the cystic duct, in approximately 90–95% of patients with AC [20]. In contrast, acalculous cholecystitis accounts for approximately 5–10% of all cases of AC. Acalculous cholecystitis usually occurs in critically ill patients receiving total parenteral nutrition; it has been reported to occur mainly in men over 50 years of age [21, 22]. Several studies have reported an increased incidence of acalculous cholecystitis in the outpatient population, including patients with atherosclerosis, as seen in hypertension and diabetes [23, 24]. In the present study, there was an increase in acalculous cholecystitis with age, which seems to be related to an increase in atherosclerotic vascular disease with age.

CBD stones, cholangitis, and gallstone pancreatitis may be present in patients with AC. In the general population, 5% of patients with cholecystitis have coexisting CBD stones [25]. The risk of gallstones increases with age regardless of ethnicity [26], and concomitant gallbladder and CBD stones have been reported to be associated with increasing age [27]. In the present study, we confirmed that the proportion of CBD stones combined with AC gradually increased with age. Therefore, older patients with AC should be checked for combined CBD stones, and an appropriate treatment plan should be established.

LC is accepted as a relatively safe procedure with a mortality rate of less than 1% [28]. However, the risk of postoperative complications increases in patients with advanced age, and recently published studies reported a mortality rate of 1.7–10.0% after LC for AC in octogenarians [10, 29, 30]. Similar to previous studies, our study also reported a mortality rate of 2.1% after LC in patients aged > 80 years. In elderly patietns with AC, ASA classification and CACI increase with increasing age and comorbidity, which can increase the risk of general anesthesia and postoperative complications. In addition, as show in our study, the severity of AC increases with age, making surgery technically challenging. In general, elderly patietns are slower to return to mobility and daily activities after surgery. Early discharge after surgery can be a social issue, especially for older patients who live alone and require daily care. These factors contribute to an increased length of hospital stay after LC in elderly patietns with AC, and indeed, in this study, we confirmed that the length of hospital stay increased with increasing age. Therefore, in the treatment of AC, older patients over 80 years of age should fully consider the risk of LC and decide to undergo surgery.

PTGBD is an alternative treatment to avoid urgent surgery for AC in high-risk or older patients. However, PTGBD is not a definitive treatment option for AC that can replace LC. We previously reported that elective LC is recommended in AC after PTGBD for patients aged > 80 years because of the high recurrence rate of biliary events after PTGBD removal and the difficulty associated with PTGBD maintenance [8]. In older patients with AC, it is important to decide on surgery by evaluating the risks and benefits of LC through a detailed preoperative evaluation rather than by avoiding LC in all cases. In the multivariate analysis of our study, BMI < 18.5 kg/m^2^, serum albumin < 2.5 mg/dL, and moderate/severe AC were statistically significant risk factors for serious complications after LC in patients aged > 80 years. It was confirmed that the severity of AC and the patient’s preoperative nutritional status had a significant impact on postoperative outcomes. Therefore, in older and malnourished patients with AC, urgent surgery should be avoided, and surgery should be performed after sufficient supportive care to restore nutritional status before LC.

In particular, in patients with AC, it is not easy to distinguish early gallbladder cancer through imaging studies in which a mass is not clearly formed due to wall thickening of the gallbladder due to inflammation. Several previous studies have reported an increased risk of incidental gallbladder cancer diagnosed after LC for benign gallbladder disease associated with advanced age [31, 32]. In the present study, it was confirmed that the risk of incidental gallbladder cancer increased with age, particularly in patients with AC (age ≥ 80 years; OR 11.713, *p* = 0.002). Therefore, in older patients with AC, the possibility of gallbladder cancer should be sufficiently explained before LC, and sufficient attention should be paid during LC to prevent the occurrence of bile spillage, which may adversely affect prognosis [33].

The present study had several limitations. First, this was a single-institutional retrospective study, and our results may have limited generalizability, although a relatively large number of patients who underwent LC were included. Second, there was a selection bias as the study included only patients who underwent surgical treatment. Finally, our study only evaluated short-term surgical outcomes. Further studies that include long-term outcomes, such as quality of life, are needed.

## Conclusion

Advanced age was associated with severe AC, worse surgical outcomes, and a higher rate of incidental gallbladder cancer following LC. Therefore, in patients over 80 years of age with AC, especially those with poor nutritional status and high severity grading, urgent surgery should be avoided, and surgery should be performed after sufficient supportive care to restore nutritional status before LC.

## Data Availability

The datasets used and/or analysed during the current study are available from the corresponding author on reasonable request.

## Abbreviations

AC: acute cholecystitis
LC: laparoscopic cholecystectomy
PTGBD: percutaneous transhepatic gallbladder drainage
CBD: common bile duct
CACI: Charlson Age Comorbidity Index
ASA PS: American Society of Anesthesiologists physical status
BMI: body mass index
PCD: percutaneous drain
